# Susceptibility of algae to Cr toxicity reveals contrasting metal management strategies

**DOI:** 10.1002/lno.11183

**Published:** 2019-04-22

**Authors:** Will Wilson, Qiong Zhang, Rosalind E. M. Rickaby

**Affiliations:** ^1^ Department of Earth Sciences University of Oxford Oxford UK

## Abstract

At the Paleozoic–Mesozoic boundary, the dominance of marine eukaryotic algae shifted from the green (chlorophyll *b*) to the red (chlorophyll *c*) superfamily. Selection pressures caused by the bioavailability of trace metals associated with increasing oxygenation of the ocean may have played a key role in this algal revolution. From a scan of elemental compositions, a significant difference in the cellular Cr/P quota was found between the two superfamilies. Here, the different responses to high levels of Cr exposure reveal contrasting strategies for metal uptake and homeostasis in these algal lineages. At high Cr(VI) concentrations, red lineages experience growth inhibition through reduced photosynthetic capability, while green lineages are completely unaffected. Moreover, Cr(VI) has a more significant impact on the metallomes of red lineage algae, in which metal/P ratios increased with increasing Cr(VI) concentration for many trace elements. Green algae have higher specificity transporters to prevent Cr(VI) from entering the cell, and more specific intracellular stores of Cr within the membrane fraction than the red algae, which accumulate more Cr mistakenly in the cytosol fraction via lower affinity transport mechanisms. Green algal approaches require greater nutrient investments in the more numerous transport proteins required and management of specific metals, a strategy better adapted to the resource‐rich coastal waters. By contrast, the red algae are nutrient‐efficient with fewer and less discriminate metal transporters, which can be fast and better adapted in the oligotrophic, oxygenated open ocean, which has prevailed since the deepening of the oxygen minimum zones at the start of the Mesozoic era.

The modern open ocean is dominated by chlorophyll *a* + *c* containing (red lineage) algae whereas the Chl *b* containing (green lineage) algae play a comparatively minor marine ecological role, being restricted to, and dominating freshwater environments (Grzebyk et al. 2003). Contrasting preference among the algal groups for these different ecological niches appears to have emerged relatively recently. Phylogenetics, algal biomarkers, and the microfossil record suggest that, although the red lineage algae can be evidenced as far back as ~ 1 Ba, the algae containing Chl *a* + *c* rose to dominance over the incumbent green algae in the open ocean ~ 200 million years ago (Tappan 1980; Knoll 1992; Butterfield, 2015, Quigg et al. 2003; Falkowski et al. 2004b, 2005; De Vargas et al. 2007; Knoll et al. 2007; Kodner et al. 2008). The physiological and ecological distinctions between the red and green lineage algae have been attributed to a number of factors ranging from the origin of the plastid to nutrient storage capacity (Quigg et al. 2003; Falkowski et al. 2004a,*b*), but no consensus has emerged.

Evidence of environmental change in the open ocean, coincident with the proliferation of the red algal lineage, may provide a clue as to which algal attribute(s) are under selection at this time. A recent compilation of I/Ca in marine carbonates shows a step‐change in oxygenation of the upper ocean and deepening of oxygen minimum zones (OMZs) to sustainable near modern conditions ~ 200 Ma (Lu et al. 2018). Significant changes in the areal extent of OMZs drove changes in the dissolved oceanic concentration of many redox sensitive trace metals. Indeed, the distinctive origin of the green and red plastids, from primary and secondary endosymbiotic origins, respectively, may have conferred contrasting trace metal requirements between these algal lineages which led to differential success during the algal radiation in the more oxygenated Mesozoic ocean (Quigg et al. 2003; Falkowski et al. 2004a; Keeling et al. 2004; Keeling 2010).

From extended algal cell chemistry (Rickaby et al. 2017; Zhang et al. 2018; Fig. 1), it has emerged that Cr could illustrate physiological contrasts between the red and green lineage algae. There is a significant difference in the uptake and storage of Cr between the green and red algal lineages such that red algae have a much higher cell quota of Cr, normalized to P, than green algae. To date, research suggests that algae have no biological use for Cr(III) or Cr(VI). Rather, Cr is nonessential and toxic to algae, when in high concentrations. Toxicity due to Cr(VI) generally arises from its reduction to Cr(III) within the cell that produces reactive oxygen species (ROS) that react with DNA and produce harmful alterations. The ROS act to inhibit cell division and therefore prevent the algae from reproducing asexually. Often residual Cr(III) remains in the cell wall when the Cr(VI) is reduced (Volland et al. 2012). Mechanisms to defend against Cr exposure have been identified; the most established is via the use of glutathione—an antioxidant that prevents damage from ROS. Glutathione can also remove Cr directly, by reacting and forming a complex (Volland et al. 2012). Some species of algae have been found to resist extremely high Cr concentrations of up to 80,000 *μ*g L^−1^ in polluted waters (Cervantes et al. 2001).

**Figure 1:**
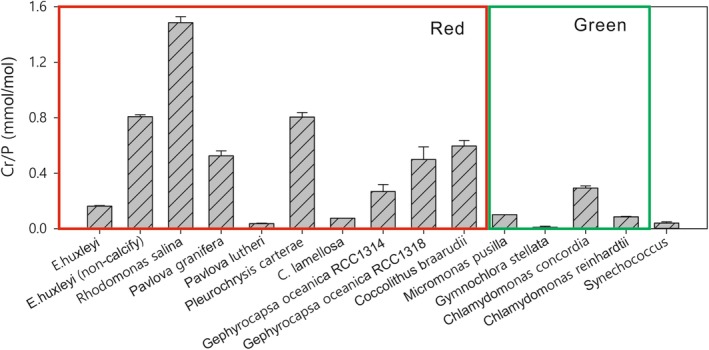
Whole cell Cr/P ratios in various phytoplankton in different superfamilies grown in unmodified Aquil* medium (except for freshwater chlorophyte C. reinhardtii which was cultured in Sueoka's high salt medium supplemented with acetate). Generally, the red superfamily has a higher Cr/P quota than the green superfamily.

No mechanisms specifically for the uptake of Cr have been discovered in algae. However, several routes of entry for Cr have been found. First, Cr uptake can occur through membrane sulfate channels (Cheung and Gu 2007). Second, uptake of Cr has been shown to occur through carriers used for the uptake of other, essential, metals for metabolism. This method of uptake is possible because Cr is able to displace these essential metals from physiologically important centers, in particular Fe (Moshtaghie et al. 1992; Pandey and Sharma 2003; Shanker et al. 2005; Macomber and Imlay 2009). The current consensus is that Cr(VI) is actively taken up using metabolic energy, whereas Cr(III) is passively taken up and/or extracellularly adsorbed and does not require metabolic energy (Shanker et al. 2005; Volland et al. 2012; Semeniuk et al. 2016).

Our preliminary evidence of a difference in cellular Cr content between red and green algal lineages, coupled with the possibility that the Mesozoic oxygenation of the ocean drove a change in oceanic Cr content, due to the relatively high oxidation potential of insoluble Cr (III) to the soluble Cr (VI), prompted us to investigate why Cr quotas are different between the red and the green algae. This study will examine the differences in physiology—growth, fluorescence, and metal uptake—between the two lineages. Although our aim was to determine whether different responses to Cr(VI) could play a role in driving the shift from the green to the red lineage in the Mesozoic, the response to Cr instead portrays the contrasting strategies of these algal lineages to metal management which may have contributed to their different success in the marine environment at the Paleozoic–Mesozoic boundary. An additional motivation for examining green vs. red algal response to increasing Cr concentrations is the potential for yielding new and enlightening data for their growing use in the bioremediation of industrial Cr pollution. Biosorption of heavy metals from industrial wastewater via plants and algae is increasingly used as a cheaper alternative to activated carbon (Vijayaraghavan and Yun 2008).

## *Methods and materials*

### Cr concentrations and growth media

Values for natural concentrations of Cr in freshwater and seawater are in good agreement with each other across different sources. Values of 0.1–500 *μ*g L^−1^ (2 nmol L^−1^–10 *μ*mol L^−1^) are reported for freshwater and of 0.2–50 *μ*g L^−1^ (4 nmol L^−1^–1 *μ*mol L^−1^) are reported for seawater (Jan and Young 1978; Hirata et al. 2000; Cervantes et al. 2001; Shanker et al. 2005; Freslon et al. 2011; Moos and Boyle 2018) with open ocean concentrations of 2.4 nmol L^−1^ (125 ng L^−1^, Jeandel and Minster 1984, Goring‐Harford et al. 2018). The concentrations of Cr in our experiments were selected to first encompass natural Cr concentrations in seawater, and second, reflect the reported concentrations at which Cr became toxic and inhibit the growth of phytoplankton in Supporting Information Table S1. Accordingly, the concentrations of Cr used were 0 *μ*g L^−1^ (control), 10 *μ*g L^−1^ (0.2 *μ*mol L^−1^), 100 *μ*g L^−1^ (2 *μ*mol L^−1^), and 1000 *μ*g L^−1^ (20 *μ*mol L^−1^).

The cultures were grown in acid clean sterile plastic culture flask. The blanks of Cr for the culture flasks were checked before the experiments and were confirmed to be beneath detection. The growth medium used was Aquil*, made according to the methods of Sunda et al. (2005). Cr was added as K_2_Cr_2_O_7_; K^+^ was buffered and the concentration did not increase in the growth media. The desired Cr concentrations were achieved by pipetting 150 *μ*L of × 1000 concentrated Cr stock solutions into 149.85 mL of the Aquil* growth medium. For the control, 150 *μ*L of Milli‐Q was used instead of chromate solution. Each 150 mL solution was then split into 3 × 50 mL to create triplicate growth solutions. Each culture was grown in triplicates to measure the replicability of the results. All growth media were adjusted to pH 8.2, the present average pH of the ocean. The growth media were sterilized by filtering through 0.22 *μ*m filters.

### Algal cultures

The algal cultures were obtained from the Roscoff Culture Collection (RCC). The representative green algae used were *Chlamydomonas concordia* (RCC1) and *Ostreococcus tauri* (RCC4221). The representative red algae used were the noncalcifying *Emiliania huxleyi* (RCC1242) and *Thalassiosira pseudonana* (RCC950). Triplicate algal cultures for each Cr concentration were produced by pipetting 1 mL aliquots of each algal culture, in the exponential growth phase, into 3 × 50 mL of the growth solution (Aquil* with the appropriate concentration of Cr added). The cultures were grown at a constant temperature of 20 ± 1°C, pH 8.2 and illuminated with 100 *μ*mol photons m^−2^ s^−1^ on a light to dark cycle of 12:12 h.

### Growth rate measurements

The population sizes of different algal species were measured either by using an electronic particle counter Coulter Counter® (for RCC1 and RCC1242) to determine the cell numbers and sizes simultaneously, or a TECAN Spark® Multimode plate reader (for all four species studied) to measure the chlorophyll fluorescence.

The growth rates for different species were calculated using the following equation:

$$N_{2}=N_{1}e^{\mu \;t}$$

where *N*_1_ is the previous population size, *N*_2_ is the current population size at a time *t* days after *N*_1_, and *μ* is the growth rate.

### Fluorescence measurements, *F*_v_/*F*_m_ and *σ*_PSII_

Fluorescence data, specifically the *F*_v_/*F*_m_ and *σ*_PSII_ measurements, give a measure of stress caused to photosystem II (PSII) due to external factors. The fluorescence data were collected using the Satlantic FIRe System fluorometer. In general, the greater the stress, the lower *F*_v_/*F*_m_ ratio. *σ*_PSII_ is the functional absorptional cross‐sectional area of PSII and indicates how efficiently PSII is harvesting light. *F*_v_/*F*_m_ and *σ*_PSII_ data were used to analyze the effect that Cr had on the photochemical reactions in PSII, and therefore quantify the level of stress that Cr placed the algae under. *F*_v_/*F*_m_ data are purely informative by comparison of treatments within an individual species. *F*_v_/*F*_m_ data were collected from second‐generation cultures of *C. concordia* and *E. huxleyi*.

### Whole cell digestion and Inductively coupled plasma mass spectrometry (ICP‐MS)

Whole cell digestions of the algae were performed to ascertain the amount of Cr being taken up both onto and into the cell, and the amount of Cr remaining in the growth media. Cr concentrations are normalized to P. Whole cell digestion data were collected from second‐ and third‐generation cultures of *C. concordia* and *E. huxleyi*. Cells were collected via centrifugation: the cells were centrifuged at 4700 rpm for 1 h. The initial supernatant was removed and saved for measurement on the ICP‐MS to quantify the amount of Cr remaining in the growth medium. The cells were then washed two times with chelexed synthetic ocean water (made according to the methods of Morel et al. 1979) and one time with Tris buffer (pH = 8.2) in order to remove the loosely bound metals on cell surfaces. The pellets were then digested in HNO_3_ and H_2_O_2_ and metal contents were measured using the ICP‐MS, according to the methods of Zhang et al. (2018).

To determine the cellular distribution of Cr, the cells were split into three procedurally defined cell fractions: (1) “Protein”—proteins and some organelles within the cytoplasm of the cell; this represents the inner organelles of the cell. (2) “Cell Debris” (CD)—the membranes that were not broken down in the removal of the protein fraction; this represents the cell wall and surface membranes. (3) “Metal rich granules” (MRG)—all insoluble material that was not broken down in the dissolution of the CD; this represents the NaOH‐resistant fraction of the membranes. The different fractions were retrieved following Aharchaou et al. (2017) and the process is briefly described as follows: first, to separate the protein fraction, the cells were disrupted by ultrasonication. During ultrasonication, the cells were placed on ice and in 1 mL of extraction buffer (20 mmol L^−1^ Tris, pH 7.4) to prevent denaturation of the proteins. Each sample was disrupted six times for 20 s bursts, at a pulse frequency of 0.4 s s^−1^, with 20 s intervals between ultrasonications. The resulting suspension was then centrifuged for 30 min at 13,200 rpm and 4°C. The supernatant, containing the protein fraction, was collected for protein analysis. Second, 0.5 mL Milli‐Q water was added to the pellet before boiling the samples for 2 min, and then 0.5 mL of 1 mol L^−1^ NaOH was added into each sample and digest the samples at 70°C for 60 min. The CD fraction could be separated from the MRG via another centrifugation for 30 min and collection of the resulting supernatant. Last, the pellet containing the MRG was digested with in‐house distilled HNO_3_ and ultrapure H_2_O_2_. The respective solutions containing the protein, CD, and MRG fractions were then measured on the ICP‐MS, again, according to the methods of Zhang et al. (2018).

## *Results*

### Growth rate

**Figure 2:**
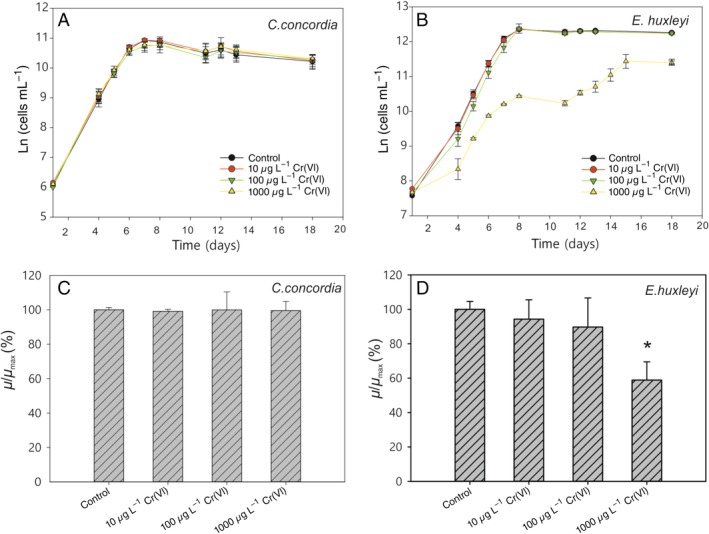
The effects of increasing Cr(VI) on cell growth of *C. concordia* and *E. huxleyi*. The growth curves of *C. concordia* and *E. huxelyi* are shown in (**A**, **B**), and the relative maximum growth rates are shown in (**C**, **D**). The exponential growth phase can be clearly identified on the growth curves where cell count rapidly increases. Maximum growth rates are taken from data within the exponential phase. *μ*_max_ is the maximum growth rate from the control group. Significant inhibition of growth was found in *E. huxleyi* under 1000 *μ*g L^−1^ Cr(VI), while no noticeable effect was found in *C. concordia* under the same conditions. Data in this figure are derived from cell numbers collected by using a coulter counter.

**Figure 3:**
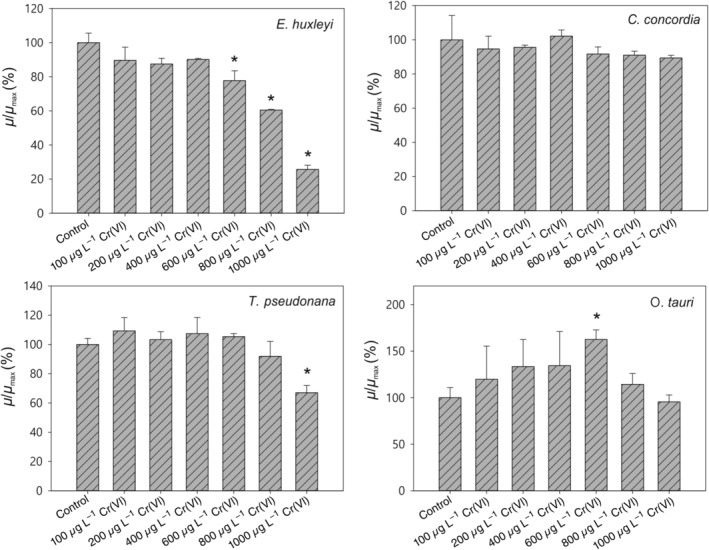
High concentrations of Cr(VI) inhibited the growth for the red algae *E. huxleyi* (> 600 *μ*g L^−1^) and T. pseudonana (1000 *μ*g L^−1^), but had no impact on the growth of the green algae *C. concordia* and *O. tauri*. Cr(VI) has nutrient‐like profile for *O. tauri*, with an optimum concentration at 600 *μ*g L^−1^. For reference, typical open ocean concentrations of Cr(VI) are ~ 125 ng L^−1^. *: *p* < 0.05. *μ*_max_ is the maximum growth rate from the control group. Data in the figure are derived from chlorophyll measurement using a plate reader.

The data reveal a strikingly different response between green lineage algae and red lineage algae to elevated Cr concentrations (Figs. 2, 3), at least 100‐fold higher than open ocean concentrations. In *C. concordia*, no significant difference in growth curves was found between the control group (no added Cr[VI]) and the groups with different Cr concentrations up to 1000 *μ*g L^−1^, suggesting *C. concordia* was not affected by Cr(VI). In *E. huxleyi*, however, the growth was significantly inhibited at 1000 *μ*g L^−1^ Cr(VI) (Fig. 3, *p* < 0.01). The growth rates of *T. pseudonana* were remarkably similar to *E. huxleyi*, with concentrations up to and including 800 *μ*g L^−1^ Cr(VI) having no significant effect on growth, but at 1000 *μ*g L^−1^ Cr(VI), the growth rate was notably suppressed (*p* < 0.01). Interestingly, the growth rates of *O. tauri* increased with Cr(VI) concentration up to 600 *μ*g L^−1^, indicating that Cr may be beneficial for this species, even acting as a nutrient up to this concentration but then becoming toxic at higher concentrations (Fig. 3). It has to be noted that, in Fig. 2, the data are derived from cell numbers measured by coulter counter, while in Fig. 3, the data are derived from chlorophyll measured by plate reader. The chlorophyll concentration/cell may have been impacted by the toxicity of Cr(VI). Therefore, the relative growth rate for *E. huxelyi* under 1000 mg L^−1^ Cr(VI) was lower in Fig. 3 than in Fig. 2. Nonetheless, the growth rate data determined by the two methods produced the same general trends for *C. concordia* and *E. huxleyi*, validating the use of chlorophyll concentration in place of cell count in the calculation of growth rates.

### Cell size

**Figure 4:**
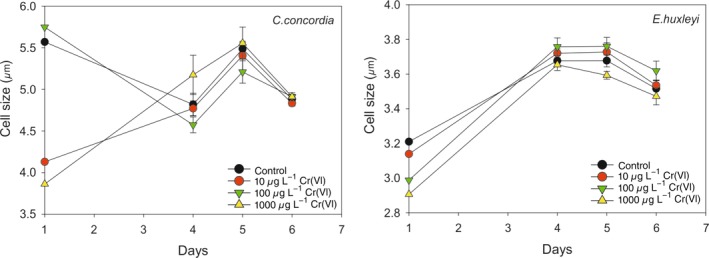
The effects of increasing Cr on cell size of *C. concordia* and *E. huxleyi* over the exponential phase.

Cell sizes for *C. concordia* and *E. huxleyi* were measured in the study, and the data are shown in Fig. 4. For *C. concordia*, the cell size data show no clear trends, i.e.*,* deviations from the control. The difference in cell sizes in the early days of the experiment may be due to broken CD. However, for *E. huxleyi*, the cell size was consistently smaller (*p* < 0.05) in the group with the highest concentration than all the other groups, indicating such high levels of Cr(VI) also impacts on the size of the cell. Note that the cells from the first day were inoculated from a previous generation of cells cultured under the same level of Cr(VI) employed in this study.

### Fluorescence

The fluorescence data were collected during the whole period of the experiments for *C. concordia* and *E. huxleyi*. In *C. concordia*, the *F*_v_/*F*_m_ curves are similar, decreasing from 0.83 on day 4 (exponential phase) to 0.66 ± 0.02 on day 8 (stationary phase). In *E. huxleyi*, the *F*_v_/*F*_m_ curves for concentrations 0, 10, and 100 *μ*g L^−1^ Cr are nearly identical over days 4–8, rising from 0.34 to 0.39 and then falling to 0.38. At 1000 *μ*g L^−1^ Cr, the *F*_v_/*F*_m_ is consistently reduced: decreasing from 0.21 on day 4 to 0.09 on days 6–8.

The *σ*_PSII_ data support the *F*_v_/*F*_m_ data. In *C. concordia*, the *σ*_PSII_ values are very similar for all Cr concentrations, *σ*_PSII_ even increases slightly under 1000 *μ*g L^−1^ Cr—suggesting that high Cr could actually improve the light harvesting efficiency of *C. concordia*. The *σ*_PSII_ of *E. huxleyi* is equivalent for all Cr concentrations up to day 7. However, on day 8, the *σ*_PSII_ of 1000 *μ*g L^−1^ Cr is 80l Å^2^ smaller than the *σ*_PSII_ values of the other Cr concentrations.

In this study, *E. huxleyi* reached early stationary phase after 6 d of culturing with Cr concentrations up to 100 *μ*g L^−1^. However, in the group with 1000 *μ*g L^−1^ Cr, both the lag phase and the exponential phase of *E. huxleyi* extended, possibly due to fewer cells in total in the medium. *E. huxleyi* reached stationary phase after 13 d of culturing at 1000 *μ*g L^−1^ Cr. Therefore, both *F*_v_/*F*_m_ and *σ*_PSII_ increased from day 8 to day 13. However, the maximum *F*_v_/*F*_m_ and *σ*_PSII_ in the group at 1000 *μ*g L^−1^ Cr was still much lower than those in the groups at lower Cr concentrations.

**Figure 5:**
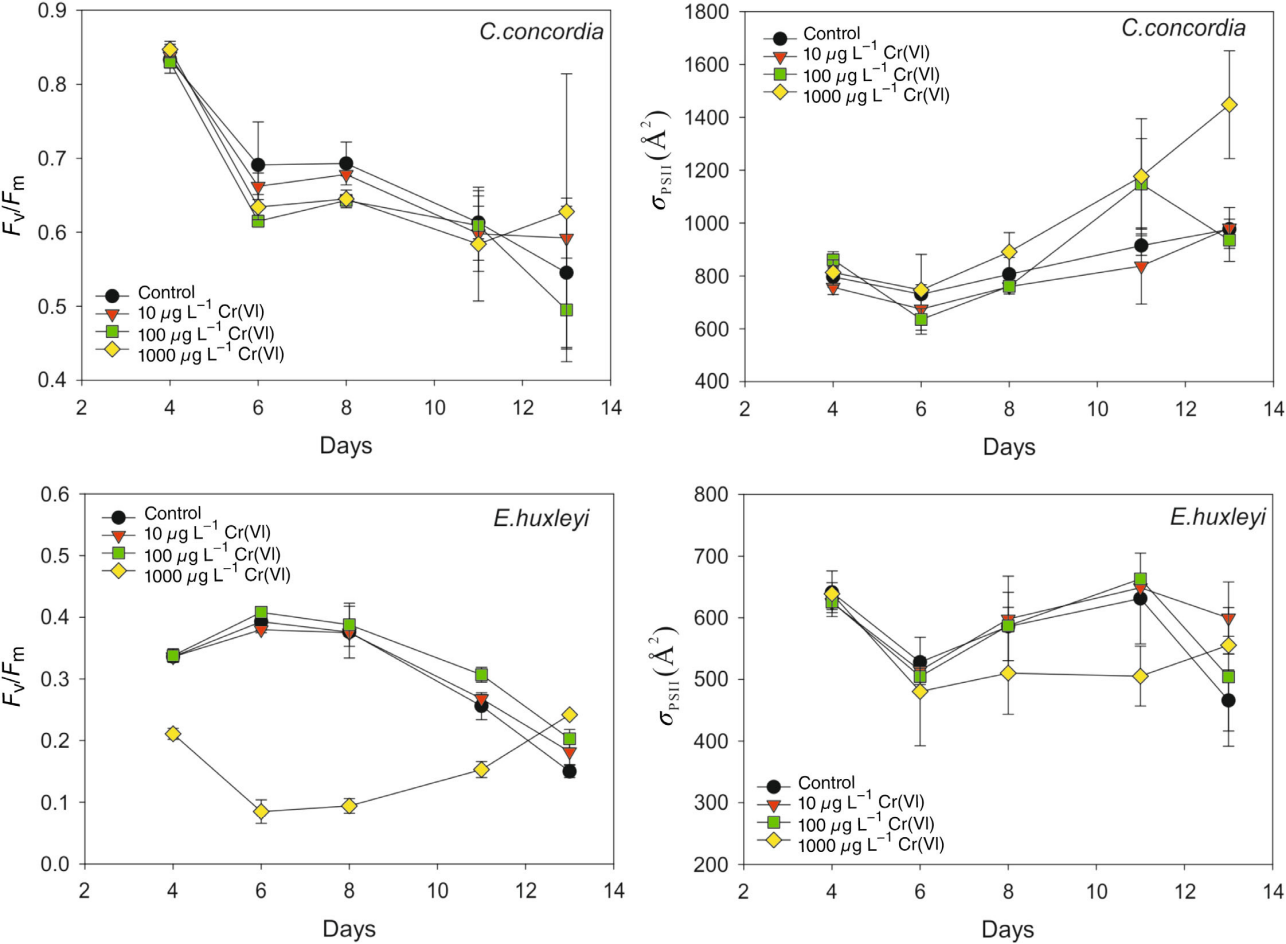
Changes to *F*_v_/*F*_m_ ratio and *σ*_PSII_ in *C. concordia* and *E. huxleyi* over the experimental period and under different Cr(VI) concentrations. Both the photochemical efficiency (*F*_v_/*F*_m_) and light‐harvesting capability (*σ*_PSII_) in *C. concordia* are unaffected by the presence of Cr(VI), but in *E. huxleyi* are significantly diminished under the highest concentration of Cr(VI).

The two fluorescence parameters (Fig. 5) together suggest that the photosynthetic capability of *C. concordia* is unaffected by the presence of Cr, whereas both the photochemical efficiency (*F*_v_/*F*_m_) and light‐harvesting capability (*σ*_PSII_) of PSII in *E. huxleyi* are significantly diminished under the highest concentration of Cr.

### Whole cell metallomes

**Figure 6:**
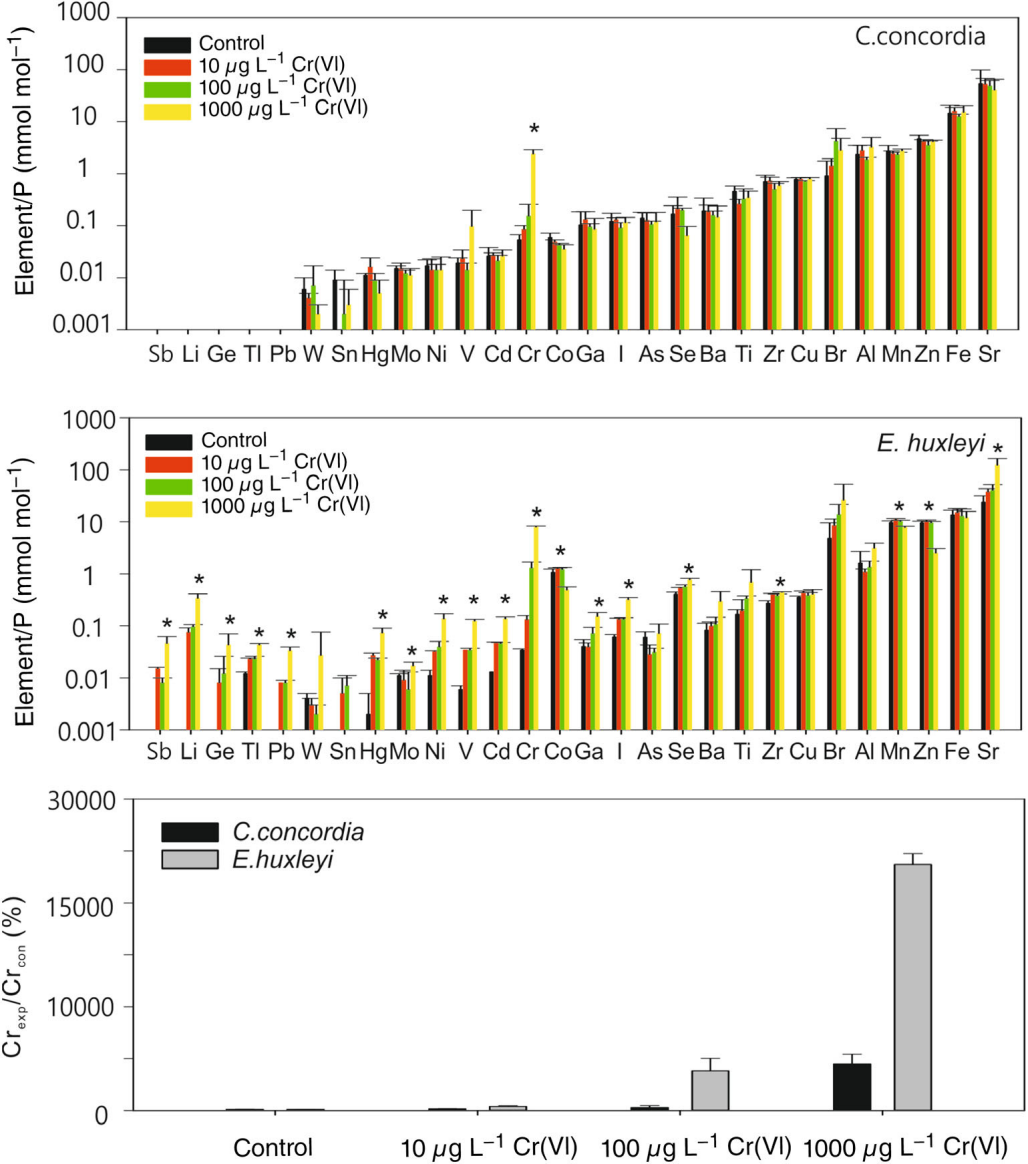
The metallome of *C. concordia* and *E. huxleyi*, i.e., the total metal content of the cell, is represented in terms of mmol mol^−1^ P for cells grown at varying Cr(VI) concentrations. A more significant increase of Cr/P was found in *E. huxleyi* than in *C. concordia* with increasing Cr(VI) concentration in the medium (bottom panel). The concentration of many other elements in *E. huxleyi* cells also significantly increases with increasing Cr(VI) concentration in the medium, except for the essential trace elements such as Mn, Fe, and Zn. Metal homeostasis is more stable in *C. concordia* than in *E. huxleyi*. *: *p* < 0.05. Cr_exp_/Cr_con_: Percentage change of Cr/P (mmol mol^−1^) in the Cr‐treated groups comparing to the control group.

The metallomes of *C. concordia* and *E. huxleyi* grown at different Cr(VI) concentrations are shown in Fig. 6. With increasing Cr(VI), the Cr/P in both species increased, but the increases in *E. huxleyi* are much larger than those in *C. concordia*. The response of these two algae to Cr(VI), in terms of their trace element contents (presented as metal/P ratio in mmol mol^−1^), are significantly different.

For *C. concordia*, the increase of Cr(VI) in the medium did not significantly impact the elemental quota. The concentrations of most elements stayed unchanged or slightly decreased with increasing Cr(VI) concentration, except for V and Br, which showed an increase.

In contrast, for *E. huxleyi*, most elemental concentrations significantly increased with increasing Cr(VI) concentration (*p* < 0.05). However, those known as bioessential elements, such as Fe, Mn, Zn, Cu, and Co, generally decreased with increasing Cr(VI) concentration.

### The distribution of intracellular Cr

**Figure 7:**
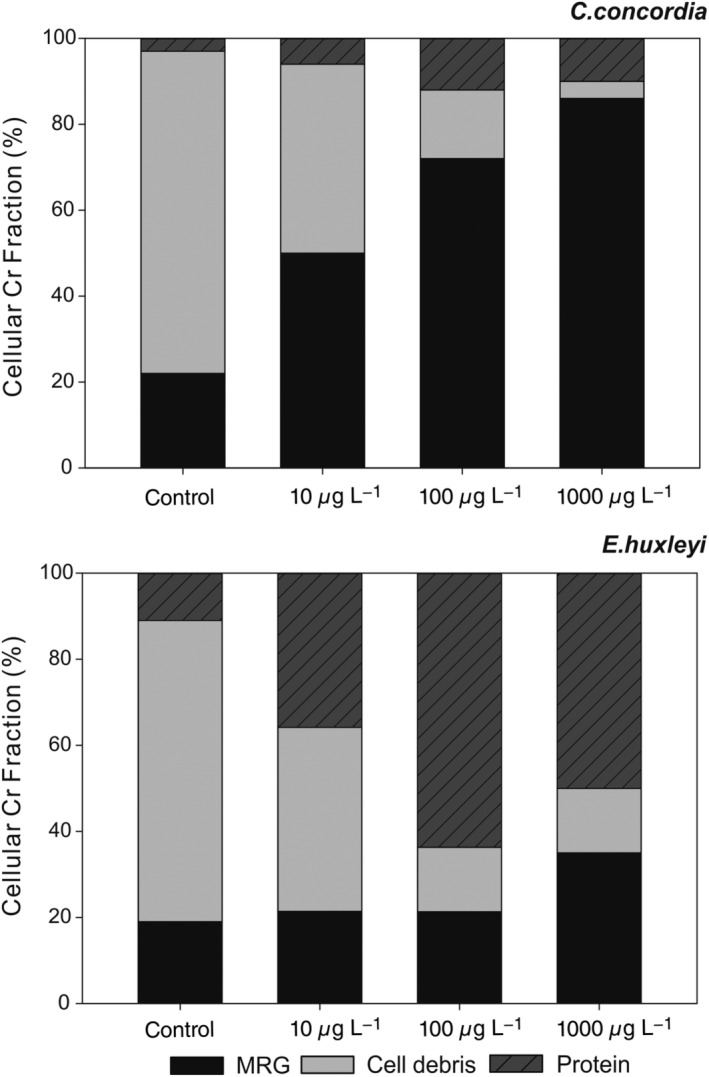
The relative proportions of Cr in the cellular fractions of *C. concordia* and *E. huxleyi* grown in 10, 100, and 1000 *μ*g L^−1^ Cr(VI). The protein fraction is representative of Cr accumulating in the inner cell. The CD and MRG fractions are representative Cr accumulating in the outer membranes of the cell.

The protein, CD, and MRG fractions in Fig. 7 are normalized to whole cell Cr to establish the relative cellular proportions of Cr accounted for by each fraction. The CD and MRG fractions are taken to represent the material from the membrane and the protein fraction is taken to represent the inner organelles of the cell.

The data reveal that in *C. concordia*, a much higher proportion of Cr accumulated in the CD and MRG fractions (0.9/1) than in the protein fraction (0.1/1) at highest Cr concentration (1000 *μ*g L^−1^), while in *E. huxleyi*, a considerably higher proportion Cr accumulated in the protein fraction (0.55 ± 0.05/1) than in *C. concordia*. This means that much more Cr accumulated in the cytosol fractions of the cell in *E. huxleyi* than in *C. concordia*. In *C. concordia*, Cr preferentially accumulated in membranes of the cell.

Normalizing the Cr data to whole cell P more reliably accounts for variations in biomass, allowing for a more quantitative comparison of Cr accumulation in the green and red algae. Table 1 shows that Cr accumulation in the protein fraction of *C. concordia* is consistently one order of magnitude lower than Cr accumulation in the protein fraction of *E. huxleyi*. Also, significantly more Cr accumulates overall in *E. huxleyi* than in *C. concordia*: at 1000 *μ*g L^−1^ Cr, *E. huxleyi* (10.20 mmol mol^−1^ P) accumulates three times as much Cr than *C. concordia* (3.38 mmol mol^−1^ P). In both algae, Cr accumulation does not increase 10‐fold with each 10‐fold increase in Cr concentration, indicating that Cr accumulation in algae is not purely a passive function of the external environment.

**Table 1:** The cellular proportions of Cr in C. concordia and E. huxleyi grown in 10, 100, and 1000 *μ*g L^−1^ Cr, normalized to whole cell P.

		*C. concordia* Cr/P (mmol mol^−1^)	*E. huxleyi* Cr/P (mmol mol^−1^)
0 *μ*g L^−1^ Cr (Control)	MRG	0.02	0.02
	CD	0.07	0.08
	**Protein**	**1.4 × 10** ^**−3**^	**0.02**
	Total	0.09	0.12
10 *μ*g L^−1^ Cr	MRG	0.08	0.04
	CD	0.07	0.09
	**Protein**	**0.008**	**0.08**
	Total	0.16	0.21
100 *μ*g L^−1^ Cr	MRG	0.36	0.20
	CD	0.08	0.14
	**Protein**	**0.05**	**0.59**
	Total	0.49	0.93
1000 *μ*g L^−1^ Cr	MRG	2.86	3.64
	CD	0.14	1.49
	**Protein**	**0.38**	**5.07**
	Total	3.38	10.20

Normalizing to whole cell P more reliably accounts for differences in biomass. The protein fraction data are highlighted in bold.

## *Discussion*

The growth rates and fluorescence results illustrate a clear difference in the physiological response of the green algae and the red algae to increasing Cr(VI) conditions, orders of magnitude above those found in the uncontaminated open ocean. For the green algae, *C. concordia* appears to be completely unaffected at all concentrations of Cr and *O. tauri* even had an enhanced growth rate at up to 600l *μ*g L^−1^ Cr(VI), whereas for the red algae, Cr(VI) poisons *E. huxleyi* at concentrations higher than 600l *μ*g L^−1^ and *T. pseudonana* at concentrations higher than 1000 *μ*g L^−1^. The effects of Cr poisoning on *E. huxleyi* are plain: it has a lower overall cell count; a delayed entry into the exponential growth phase; a slower growth rate; and a lower fluorescence response, seen in both *F*_v_/*F*_m_ and *σ*_PSII_, indicative of stress resulting from at least chemical alteration to the PSII.

There are many potential reasons for the difference in the physiological response of the two algal lineages to increased Cr(VI): differences in cell membrane characteristics, transport mechanisms, number of sulfate channels (through which Cr(VI) can pass), detoxification mechanisms, and heavy metal adaptability. Here, we will explore two possible explanations for the red algae's much stronger susceptibility to Cr toxicity:

1. The green algae are preventing Cr from entering the cell in the first place, suggesting a more selective metal uptake than the red algae.
2. The green algae have superior detoxification mechanisms than the red algae.

The cell quota and cell component data provide evidence for both hypothesis (1)—that the green has a more selective uptake—and hypothesis (2)—that the green has superior detoxification mechanisms. In *C. concordia*, a much higher proportion of Cr was bound in the membrane, especially the MRG fractions, than in the intracellular protein fraction, indicative of selective uptake, as Cr is being prohibited to some extent from entering the cell. In *E. huxleyi*, more of the Cr accumulated in the inner cell, in the cytosol fraction, than in the CD and MRG fractions, symptomatic of a more indiscriminate uptake mechanism. In all of the cultures, Cr accumulation in the protein fraction of *E. huxleyi* was consistently 10 times greater than in the protein fraction of *C. concordia*. This could be indicative of a less selective uptake and an inferior detoxification mechanism in the red algae, as the green algae are preventing intracellular Cr accumulation, or expelling more Cr after it was taken up into the cell. *C. concordia* accumulated less total Cr per cell than *E. huxleyi*, again suggestive of both a more selective uptake and better detoxification mechanisms.

Explanation (1) does not preclude explanation (2), and both have the potential to shed light on the great disparity between the marine governance of green and red algae. If the greens have a more selective metal uptake than the reds, then the reds could be more suited to nutrient limited areas (the oligotrophic open ocean), as a less selective uptake would allow more metals, useful or otherwise, to enter the cell at a lower energetic cost. On the other hand, in nutrient rich areas, such as the coast where metals flush into the system from the continents and with susceptibility to suboxia and greater metal availability (Mawji et al. 2015), the reds’ advantageous ability to flush more metals through the cell would then become detrimental. The reds would be much more susceptible to poisoning by allowing too many toxic metals into the cell. If the greens have better detoxification mechanisms, they could have filled the ecological niche of the freshwater environment, where nutrients but also toxins are rich in supply. Indeed, the maximum concentration of Cr in freshwater is 500 *μ*g L^−1^, whereas it is only 50 *μ*g L^−1^ in seawater (Jan and Young 1978; Cervantes et al. 2001; Shanker et al. 2005).

Homeostatic mechanisms control the balance between poisoning due to metal excess, and enzyme inactivity due to metal deficiency (Blaby‐Haas and Merchant 2012). The metallome data in this study clearly revealed that the green alga *C. concordia* exerts more homeostatic control on its intracellular environment than the red alga *E. huxleyi*. Therefore, examining the specific homeostatic mechanisms of the two algae can further test hypotheses (1) and (2). Transmembrane protein metal transporters control homeostatic mechanisms; they act as the first line of defense to external increased heavy metal concentration; and are also critical in detoxification (Blaby‐Haas and Merchant 2012). So, investigating the proposed homeostatic mechanisms in (1) and (2) requires an analysis and comparison of their respective protein metal transporters. Analyzing metal transporters is notoriously difficult, especially in algae. No research has yet been published on the respective metal transporters of *C. concordia* (green) and *E. huxleyi* (red). Hanikenne et al. (2005) conducted a comprehensive inventory and assessment of all of the metal transporters in one green alga, *Chlamydomonas reinhardtii*, and one red alga, *Cyanidioschizon merolae* that could prove to be useful analogues to *C. concordia* and *E. huxleyi*, respectively. They found that the green lineage alga has almost doubled the number of metal transporters (41) than the red (25). The two transporters of particular significance in the study were: the Zrt‐, Irt‐like proteins (ZIP), which mediate the influx of metal cations from outside the cell into cytoplasm; and the multidrug resistance‐associated proteins (MRP), which are glutathione pumps.

The green alga has significantly more ZIP (14 in *C. reinhardtii* and only 4 in *C. merolae*), which help to prevent metals from entering the cell, suggesting more selective metal uptake in the green. The green also has more MRP (7 in *C. reinhardtii*, 2 in *C. merolae*) and therefore more glutathione pumps. Glutathione is a phytochelatin widely used in algal heavy metal detoxification mechanisms, as it can complex with heavy metals directly, and also absorb and destroy the harmful ROS that heavy metals create within the cell (Volland et al. 2012). So, the presence of more MRP in the green suggests that the green is better equipped for detoxification than the red. The subcellular distribution of Cr found in this study also confirmed that Cr in the green algae was mostly stored in the MRG, which have been shown to be a defensive mechanism for organisms compared to the higher Cr fraction from the cytosol in the red algae *E. huxleyi*. Metal transporters that are particularly specific will only transport one metal, and less specific transporters will transport more than one metal. Blaby‐Haas and Merchant (2012) found that *C. merolae* had no transporters capable of exclusively transporting one type of metal. It should be noted that *C. merolae* has a particularly abnormal habitat: sulfur‐rich acidic hot springs, so its use as an analogue for all reds is questionable. Nonetheless, if *C. reinhardtii* and *C. merolae* are representative of greens and reds in general, then Hanikenne et al.’s and Blaby‐Haas and Merchant's findings on metal transporters provide evidence that both (1) and (2) may be supported; that is, greens have more selective metal transporters than reds, and greens are also more proficient at detoxifying metals within the cell interior. Just from the number of expressed transporters between these lineages, it is clear that the green algae make a greater investment of resource in selective metal transport and detoxification than the more nutrient streamlined strategy of the red algae to express fewer and less specific transport proteins. It is likely this trade‐off between nutrients required for multiple transporters and controlled metal homeostasis vs. the nutrient‐lean strategy of transporting multiple ions less discriminately to fulfill metal requirements which dictates the ecological success of the green algae in the coastal/freshwater zone vs. the red algal success in the oligotrophic and toxin depleted open ocean.

The differential photosynthetic outputs of *C. concordia* and *E. huxleyi* under elevated Cr conditions provide additional insight into the metal handling by the different algal lineages. The fluorescence results show that *E. huxleyi*’s PSII is significantly stressed by high concentrations of Cr (Fig. 5) in comparison to the unaffected *C. Concordia*. Similarly, the metallome shows that under elevated Cr(VI) exposure, Mn is depleted in the cell quota of *E. huxleyi,* whereas many other nonessential elements, such as Ni and Sr for example, were increased. No significant changes were observed in the cell metal quota (except for Cr) of *C. concordia* (Fig. 6).

Mn is critical to the redox behavior of PSII, as it is used in water‐splitting reactions essential for photosynthesis (Blaby‐Haas and Merchant 2012; Fischer et al. 2015). In *E. huxleyi* as Cr increases within the cell, Mn content decreases, *F*_v_/*F*_m_ decreases, and *σ*_PSII_ decreases, suggesting that Mn is in some way linked to the decrease of photosynthetic output of PSII in *E. huxleyi*. Accordingly, it is plausible that in addition to toxicity due to DNA alteration by ROS, Cr toxicity may also result in reduced photosynthetic capability through Mn depletion. The D1 protein, a subunit of PSII, forms part of the water‐splitting system of PSII and coordinates the crucial Mn cluster that is utilized in light‐dependent photosynthesis (Burnap 2004; Nelson and Ben‐Shem 2004). Increasing Cr leads to pronounced decreases in the abundance of the D1 protein. Ali et al. (2006) reported that ~ 10 mg L^−1^ Cr caused a 50% decrease of the D1 protein content in *Lemna gibba* (duckweed similar to green algae). Hörcsik et al. (2007) reported that *Chlorella pyrenoidosa* experienced a 50% decrease of D1 after 72 h in 20 mg L^−1^ Cr. So, it is highly likely that Cr disrupts *E. huxleyi* by depleting the D1 protein and Mn content of PSII. As a result, *E. huxleyi* experiences a physiology characteristic of Mn‐depleted conditions, and so may upregulate its low specificity metal uptake systems to enhance metal uptake, resulting in the increased cell content of many of the nonessential metals. Due to its superior exclusion of toxic metals, *C. concordia* experiences little change under such high Cr conditions.

The notion that different complements of metal transporters between the red and green lineages played a role in the shift in dominance from green to red algae at the Paleozoic–Mesozoic boundary is not contrary to the hypothesis that differences in their endosymbiotic history led to their differential ecological niches. Different metal transporter assemblages have different proficiencies in mediating heavy metal toxicity. The host cell will add the endosymbiont's metal transporters to its original metal transporter arsenal after a successful engulfment (Blaby‐Haas and Merchant 2012). Therefore, either the separate origination of plastids in green and red algae could have led to the difference in metal transporters that control heavy metal uptake and detoxification, or the transporters of the heterotrophic host were different prior to the endosymbiotic events.

At the start of the Mesozoic, the deepening of the OMZs could have reduced a major resupply conduit of many trace metals and phosphate (remobilized in those anoxic waters) back to the surface ocean, and heralded a period of increasing limitation of major and minor nutrients in the open ocean. The nutritionally lean red algae, with their smaller numbers of low specificity but high turn‐over transporters, were well adapted to such limiting conditions without risk of excess accumulation of elements. Furthermore, we speculate that this poor specificity transport of the red lineage algae and likely upregulation of cellular metal import in the depleted Mesozoic surface ocean may have contributed to the initiation and success of calcium carbonate formation in this lineage. Other unintended metals, such as Ca, accumulate in the cell through low specificity transporters and have to be managed safely to keep cytosolic concentrations < 10^−7^ mol L^−1^, by expulsion in mineral form. The enhanced accumulation of Sr, a chemical analogue for Ca, under Cr exposure, supports this contention. Trace metal limitation is known to enhance calcification in coccolithophores (Schulz et al. 2004).

Last, since *E. huxleyi* accumulates three times as much Cr as *C. concordia*, and assuming that they are representative of the red and green lineages as a whole, future bioremediation research could profit from considering the use of algae from the red superfamily.

## *Conclusions*

The fossil record indicates that red algae have dominated the oceans since at least the start of the Mesozoic era. Exposing a representative green alga, *C. concordia*, and a representative red alga, *E. huxleyi*, to Cr concentrations at least two orders of magnitude above open ocean values, has revealed contrasts in their metal management strategies that may have played a role in the red lineage's rise to dominance in the modern open ocean. The differential ability to grow under elevated Cr conditions, and the higher Cr quota within the red lineage algae highlights a different strategic approach between these lineages to manage their metal homeostasis. The green algae exert a greater nutrient requirement to keep their cells “clean” and use a large number of metal specific transport and control system. By contrast, the nutrient lean red algal lineage is easily “dirtied” by concentrated media, since they have fewer and less specific transportation systems which are well adapted to fast transport in the chemically and nutritionally deficient areas of the open ocean, which have prevailed since the start of the Mesozoic.

## Conflict of Interest

None declared.

## Supporting information


**Table S1:** Toxic concentrations of Cr[VI] for green and red algae
**Table S2:** Cr concentration in the medium after culturing phytoplankton
