# Supplementary material for: Susceptibility of algae to Cr toxicity reveals contrasting metal management strategies
Source: Limnol Oceanogr. 2019 Apr 22;64(5):2271–82. doi: 10.1002/lno.11183 (PMC6774333; doi:10.1002/lno.11183)
Supplement: Supplementary file 1 — Table S1: Toxic concentrations of Cr[VI] for green and red algae Table S2: Cr concentration in the medium after culturing phytoplankton [file LNO-64-2271-s001.docx]

Table S1 Toxic concentrations of Cr(VI) for green and red algae

| **Algal Species** | **Green/Red** | **Fresh/Marine** | **Toxic [Cr(VI)] (μgL^-1^)** | **Effects** | **Reference** |
| --- | --- | --- | --- | --- | --- |
| *Chlamydomonas reinhardtii* | Green | Fresh | 500 | Growth inhibition | Rodríguez *et al*., 2007 |
| *Chlorella pyrenoidosa* | Green | Fresh | 20 | Growth reduced | Schroll,  1978 |
| *Chlorella pyrenoidosa* | Green | Fresh | 20 000 | Lethal | Hörcsik *et al*., 2006 |
| *Chlorella sp.* | Green | Fresh | 1000 | Growth inhibition | Filip *et al*., 1979 |
| *Chlorella vulgaris* | Green | Fresh | 10 000 | Growth inhibition | Petria, 1978 |
| *Chlorella vulgaris* | Green | Fresh | 45 000 | Lethal | Travieso *et al*., 1999 |
| *Cladophora glomerata* | Green | Fresh & Marine | 250 | Growth inhibition | Bharti, 1979 |
| *Glaucocystis*  *nostochinearum* | Green | Fresh | 1000 | Growth inhibition | Rai *et al*., 1992 |
| *Micrasterias*  *denticulata* | Green | Fresh | 250 | Growth  Inhibition | Volland *et al*., 2012 |
| “Natural phytoplankton” | Green | Marine | 100 | Growth  Inhibition | Wong & Trevors, 1988 |
| *Scenedesmus acutus* | Green | Fresh | 15 000 | Lethal | Travieso *et al*., 1999 |
| *Scenedesmus sp.* | Green | Fresh | 1000 | Growth inhibition | Filip *et al*., 1979 |
| *Selenastrum capricornutum* | Green | Fresh | 600 | Growth inhibition | Michnowicz  & Weaks, 1984 |
| *Stigeoclonium tenue* | Green | Fresh | 250 | Growth inhibition | Bharti, 1979 |
| *Ulothrix fimbriata* | Green | Fresh | 150 | Growth inhibition | Bharti, 1979 |
| *Chondrus crispus* | Red | Marine | 520 | Growth inhibition | Baumann *et al*., 2009 |
| *Palmaria palmata* | Red | Marine | 520 | Growth inhibition | Baumann *et al*., 2009 |
| *Polysiphonia lanosa* | Red | Marine | 520 | Growth inhibition | Baumann *et al*., 2009 |
| *Skeletonema costatum* | Red | Marine  (& estuarine) | 980 | Growth inhibition | Frey *et al*., 1983 |
| *Thalassiosira pseudonana* | Red | Marine  (& estuarine) | 20 | Growth inhibition | Frey *et al*., 1983 |

Table S2: Cr concentration in the medium after culturing phytoplankton

| Groups | Cr concentration (µg/L) |
| --- | --- |
| *C. concordia* control | N.D. |
| *C. concoridia* 10 µg/L Cr | 9.2 ± 0.3 |
| *C. concoridia* 100 µg/L Cr | 89 ± 36 |
| *C. concoridia* 1000 µg/L Cr | 986 ± 59 |
| *E. huxleyi* control | N.D. |
| *E. huxleyi* 10 µg/L Cr | 9.7 ± 1.2 |
| *E. huxleyi* 100 µg/L Cr | 99 ± 4 |
| *E. huxleyi* 1000 µg/L Cr | 984 ± 39 |

N.D. not detectable

*References:*

Baumann, H.A., Morrison, L. and Stengel, D.B., 2009. Metal accumulation and toxicity measured by PAM—chlorophyll fluorescence in seven species of marine macroalgae. *Ecotoxicology and Environmental Safety*, *72*(4), pp.1063-1075.

Bharti, A., Pandey, G.N. and Saxena, R.P., 1979. Physiological imbalances due to hexavalent chromium in freshwater algae [India]. *Indian Journal of Environmental Health (India)*.

Filip, D.S., Peters, V.T., Adams, E.D. and Middlebrooks, J., 1979. Residual heavy metal removal by an algae-intermittent sand filtration system. *Water Research*, *13*(3), pp.305-313.

Frey, B.E., Riedel, G.F., Bass, A.E. and Small, L.F., 1983. Sensitivity of estuarine phytoplankton to hexavalent chromium. *Estuarine,* *Coastal and Shelf Science*, 17(2), pp.181-187.

Hörcsik, Z., Oláh, V., Balogh, Á., Mészáros, I., Simon, L. and Lakatos, G., 2006. Effect of chromium (VI) on growth, element and photosynthetic pigment composition of Chlorella pyrenoidosa. Acta Biologica Szegediensis, 50(1-2), pp.19-23.

Michnowicz, C.J. and Weaks, T.E., 1984. Effects of pH on toxicity of As, Cr, Cu, Ni and Zn to Selenastrum capricornutum Printz. *Hydrobiologia*, *118*(3), pp.299-305.

Petria, V., 1978. Effect of chromium salts from water sediments on physiological processes in the alga Chlorella vulgaris. *Rev. Roum. Biol., Ser. Biol. Veg*, *23*, pp.55-57.

Rai, U.N., Tripathi, R.D. and Kumar, N., 1992. Bioaccumulation of chromium and toxicity on growth, photosynthetic pigments, photosynthesis, in vivo nitrate reductase activity and protein content in a chlorococcalean green alga Glaucocystisnostochinearum Itzigsohn. *Chemosphere*, *25*(11), pp.1721-1732.

Rodríguez, M.C., Barsanti, L., Passarelli, V., Evangelista, V., Conforti, V. and Gualtieri, P., 2007. Effects of chromium on photosynthetic and photoreceptive apparatus of the alga Chlamydomonas reinhardtii. *Environmental research*, *105*(2), pp.234-239.

Schroll, H., 1978. Determination of the absorption of Cr+ 6 and Cr+ 3 in an algal culture of Chlorella pyrenoidosa using 51 Cr. *Bulletin of environmental contamination and toxicology*, *20*(1), pp.721-724.

Travieso L. Cañizarez R.O. Borja R. Benítez F. Domínguez A.R. Dupeyrón R. Valiente V. (1999) Heavy metal removal by microalgae. *Bull. Environ. Contam. Toxicol.* 62, 144–151.

Volland, S., Lütz, C., Michalke, B. and Lütz-Meindl, U., 2012. Intracellular chromium localization and cell physiological response in the unicellular alga Micrasterias. *Aquatic toxicology*, *109*, pp.59-69.

Wong, P.T. and Trevors, J.T., 1988. Chromium toxicity to algae and bacteria. *Chromium in the natural and human environments*, pp.305-315.
